# Ultra-sensitive AAV capsid detection by immunocapture-based qPCR following factor VIII gene transfer

**DOI:** 10.1038/s41434-021-00287-1

**Published:** 2021-08-23

**Authors:** Krystal Sandza, Annie Clark, Elli Koziol, Hassibullah Akeefe, Fan Yang, Jennifer Holcomb, Kathryn Patton, Kevin Hammon, Nina Mitchell, Wing Y. Wong, Stephen J. Zoog, Benjamin Kim, Joshua Henshaw, Christian Vettermann

**Affiliations:** BioMarin Pharmaceutical, Inc., Novato, CA USA

**Keywords:** Biomarkers, Genetic techniques, Haematological diseases

## Abstract

Adeno-associated virus (AAV)-based gene therapy vectors are replication-incompetent and thus pose minimal risk for horizontal transmission or release into the environment. In studies with AAV5-FVIII-SQ (valoctocogene roxaparvovec), an investigational gene therapy for hemophilia A, residual vector DNA was detectable in blood, secreta, and excreta, but it remained unclear how long structurally intact AAV5 vector capsids were present. Since a comprehensive assessment of vector shedding is required by regulatory agencies, we developed a new method (termed iqPCR) that utilizes capsid-directed immunocapture followed by qPCR amplification of encapsidated DNA. The limit of detection for AAV5 vector capsids was 1.17E+04 and 2.33E+04 vg/mL in plasma and semen, respectively. Acceptable precision, accuracy, selectivity, and specificity were verified; up to 1.00E+09 vg/mL non-encapsidated vector DNA showed no interference. Anti-AAV5 antibody plasma concentrations above 141 ng/mL decreased AAV5 capsid quantification, suggesting that iqPCR mainly detects free capsids and not those complexed with antibodies. In a clinical study, AAV5-FVIII-SQ capsids were found in plasma and semen but became undetectable within nine weeks after dose administration. Hence, iqPCR monitors the presence and shedding kinetics of intact vector capsids following AAV gene therapy and informs the potential risk for horizontal transmission.

## Introduction

Many adeno-associated virus (AAV)-based gene therapies are currently in clinical development, including gene therapies for the bleeding disorders hemophilia A and B [1–7]. Hemophilia A is a genetic disorder caused by mutations in the *f8* gene that lead to deficient activity of coagulation factor VIII (FVIII). This protein is part of a critical step in the blood-clotting pathway and, consequently, medical treatment is essential for severe hemophilia A patients [8, 9]. AAV5-FVIII-SQ (valoctocogene roxaparvovec, BMN 270) is an AAV serotype 5 (AAV5)-based gene therapy designed to transduce liver cells, leading to the endogenous production of the SQ version of human B-domain deleted FVIII (FVIII-SQ), which increases plasma FVIII activity [1, 2, 10, 11].

After gene therapy administration, the presence of the AAV5-FVIII-SQ vector was monitored in various human biofluids, using a conventional qPCR method that tests for total vector DNA in extracted clinical specimens. Interestingly, residual vector DNA remained detectable in blood and semen for several months during follow-up monitoring [1, 2]. Further investigations showed that some of the longest-lived residual vector DNA was contained in white blood cells (peripheral blood mononuclear cells, PBMCs) that had been transduced at a low rate with the vector [2]. Most vector DNA in PBMCs was processed as is typical for AAV vectors, including re-synthesis of full-length vector genomes and fusion of inverted terminal repeats (ITRs), indicative of circular episomes [2]. Given that processing of vector DNA requires prior uncoating from the AAV5 capsid and transport to the nucleus, processed vector DNA poses only a negligible risk for horizontal transmission.

**Table 1:** Standard curve performance.

	Intra-curve precision (%CV)	Intra-curve accuracy (%RE)	Inter-curve precision (%CV)	Inter-curve accuracy (%RE)	*R*-squared	Slope
Plasma	≤23.4%	−38.6 to +42.9%	≤14.3%	−8.6 to +5.0%	>0.994	−3.54 to −3.31
Semen	≤28.4%	−45.9 to +92.1%	≤26.3%	−14.7 to +29.3%	>0.985	−3.57 to −3.37

**Table 2:** Precision, accuracy, and range of quantification in plasma and semen.

QC level	Parameter	Plasma				Semen			
		*n*	%CV	%RE	%TE	*n*	%CV	%RE	%TE
LLOQ (2.08E+05 vg/mL)	Intra-batch	2.82^+^	10.3	+8.0	18.3	3	7.9	−11.0	18.9
	Inter-batch	11	28.9	+10.4	39.3	9	14.9	−11.0	25.8
LQC (2.08E+06 vg/mL)	Intra-batch	3	16.1	−1.7	17.8	3	3.9	+0.8	4.7
	Inter-batch	12	28.4	−1.7	30.1	9	16.5	+0.8	17.2
MQC (2.08E+07 vg/mL)	Intra-batch	3	13.5	−6.6	20.1	3	9.6	−6.7	16.3
	Inter-batch	12	24.9	−6.6	31.5	9	12.6	−6.7	19.3
HQC (2.08E+09 vg/mL)	Intra-batch	3	24.1	+4.1	28.2	3	2.8	+6.6	9.5
	Inter-batch	12	14.7	−6.2	20.9	9	5.6	+6.6	12.2
ULOQ (2.08E+10 vg/mL)	Intra-batch	3	12.4	+4.1	16.5	3	7.0	+60.0	67.0
	Inter-batch	12	24.1	+4.1	28.2	9	60.4^++^	+60.0^++^	120.3^++^

^+ ^One value removed by Q*-*test.

^++ ^ULOQ of 2.08E+10 vg/mL was not be implemented. ULOQ was adjusted to 2.08E+09 vg/mL (HQC).

**Table 3:** Summary of method performance characteristics.

Parameter	Target performance criteria	Plasma	Semen
Standard curve performance	RE: −50 to +100%; CV ≤ 30%. R-squared should have been greater than 0.98. Slope of the standard curve should have been between −3.60 and −3.10.	RE: −38.6 to +42.9%; CV ≤ 23.4%. *R*^2^ ≥ 0.995. Slope: −3.54 to −3.31	RE: −45.9 to +92.1%; CV ≤ 28.4%. *R*^2^ ≥ 0.984. Slope: −3.57 to −3.37
Precision and accuracy (ANOVA)	RE: −50 to +100%; CV ≤ 30%; TE ≤ 130%	RE: −6.6 to +10.4%; CV ≤ 28.9%; TE ≤ 39.3%	RE: −11.0 to +6.6%; CV ≤ 16.5%; TE ≤ 25.8%^+^
Limit of detection (LOD)	The LOD is reported as the lowest AAV5 capsid concentration detected with 95% consistency, based on logistic regression.	LOD = 1.17E+04 vg/mL	LOD = 2.33E+04 vg/mL
		95% confidence limits: 9.05E+03 to 1.87E+04 vg/mL	95% confidence limits: 1.83E+04 to 3.88E+04 vg/mL
Selectivity	≥80% of spiked donors should show RE: −50 to +100% and CV (BCC) ≤ 30% at HQC and LLOQ. >80% of unspiked donors should be BLD	80% of donors spiked at HQC, 80% of donors spiked at LLOQ, and 90% of unspiked donors met target performance criteria.	80% of donors spiked at HQC, 100% of donors spiked at LLOQ, and 80% of unspiked donors met target performance criteria.
Specificity/interference	RE: −50 to +100% and NQC should remain BLD in the presence of non-encapsidated FVIII-SQ DNA.	No interference or non-specific detection was observed in the presence of up to 1.00E+09 vg/mL non-encapsidated FVIII-SQ DNA.	
Antibody tolerance	RE: −50 to +100% and CV (BCC) ≤ 30% in the presence of anti-AAV5 antibodies.	The method tolerated up to 141 ng/mL rabbit polyclonal anti-AAV5 serum (concentration in ADK5a equivalents)	Not assessed in this matrix

^+ ^ULOQ data not included; did not meet target criteria and values above HQC were removed from the reportable range.

**Table 4:** Capsid kinetics in plasma following AAV5-FVIII-SQ administration.

Dose (vg/kg)		No. (%) of detectable subjects	Peak conc. (vg/mL)	Time to peak conc. (weeks)	Time to last detectable sample (weeks)	No. (%) of subjects that achieved 3 consecutive negatives	Time to first negative sample confirmed by 2 consecutive samples (weeks)
6E12 (*n* = 1)		1 (100%)	BLQ^+^	1.0	2.0	1 (100%)	3.0
2E13 (*n* = 1)		1 (100%)	5.2E+05	1.2	1.2	1 (100%)	2.5
4E13 (*n* = 6)	Min	6 (100%)	4.4E+05	0.4	1.8	6 (100%)	3.0
	Median		1.9E+08	0.4	2.6		3.6
	Max		9.8E+08	1.1	8.1		9.0
6E13 (*n* = 7)	Min	7 (100%)	7.2E+05	0.4	2.0	7 (100%)	3.0
	Median		2.8E+08	0.4	2.0		3.1
	Max		1.0E+09	1.1	3.0		4.0
All subjects (*n* = 15)	Min	15 (100%)	BLQ^+^	0.4	1.2	15 (100%)	2.5
	Median		2.8E+07	0.4	2.1		3.1
	Max		1.0E+09	1.2	8.1		9.0

^+ ^*BLQ* below the limit of quantitation.

**Table 5:** Capsid kinetics in semen following AAV5-FVIII-SQ administration.

Dose (vg/kg)		No. (%) of detectable subjects	Peak conc. (vg/mL)	Time to peak conc. (weeks)	Time to last detectable sample (weeks)	No. (%) of subjects that achieved 3 consecutive negatives	Time to first negative sample confirmed by 2 consecutive samples (weeks)
6E12 (*n* = 1)		1 (100%)	2.9E+05	1.0	1.0	1 (100%)	2.0
2E13 (*n* = 1)		1 (100%)	BLQ^+^	0.4	1.3	1 (100%)	2.5
4E13 (*n* = 6)	Min	6 (100%)	BLQ^+^	0.4	0.4	6 (100%)	2.1
	Median		7.6E+05	0.6	0.9		3.6
	Max		4.8E+06	1.0	2.1		5.9
6E13 (*n* = 7)	Min	7 (100%)	2.6E+05	0.4	1.3	7 (100%)	1.8
	Median		7.7E+05	1.0	3.0		4.1
	Max		6.3E+07	1.1	8.0		9.0
All subjects (*n* = 15)	Min	15 (100%)	BLQ^+^	0.4	0.4	15 (100%)	1.8
	Median		6.6E+05	0.6	1.3		3.3
	Max		6.3E+07	1.1	8.0		9.0

^+ ^*BLQ* below the limit of quantitation.

It remained unclear, however, how long after dose administration, structurally intact and thus potentially transduction-competent AAV5 vector capsids were present in blood or seminal fluid. This may be a much shorter time than the duration during which total vector DNA is detectable by conventional qPCR since conventional qPCR does not distinguish between encapsidated and non-encapsidated (e.g. processed) forms of vector DNA. As such, conventional qPCR results do not unambiguously indicate a potential transmission risk [12]. Since a primary purpose of monitoring clinical vector shedding is to assess the risk of horizontal transmission [13–17], it became necessary to develop a novel method for the specific detection of potentially transduction-competent vectors (i.e., structurally intact vector consisting of both AAV5 capsid and encapsidated FVIII-SQ vector DNA). Cell-based transduction assays would be ideal to answer this question; however, our preliminary experiments indicated that assay sensitivity would fall short of revealing any meaningful insights, given the observed low residual vector DNA levels in the blood and shedding matrices (<1E+10 vg/mL) [1]. ELISA-based AAV capsid detection methods do exist but with similar restrictions on assay sensitivity [18, 19]. We, therefore, sought to develop a novel AAV capsid detection method that would have comparable sensitivity to conventional qPCR methods used for measuring total vector DNA in clinical shedding studies [1, 2]. To this end, we utilized the format of immunocapture-based qPCR (iqPCR), described previously to detect intact adenovirus particles, microbial antigens, and cytokines [20–24]. The method described herein capitalizes on the high target specificity of monoclonal antibodies directed against the AAV5 capsid and the exceptional sensitivity offered by qPCR detection of encapsidated vector DNA. Additional method optimization was achieved by establishing minimally required test sample dilutions, implementing sample liquefaction or cell lysis steps, and removing non-encapsidated vector DNA using Benzonase [25].

## Materials and methods

### Plasma and semen samples used for method characterization

Plasma samples were collected from hemophilia A donors using sodium citrate as an anticoagulant. Semen samples were collected from non-disease state male donors and stored frozen within one hour of collection. Pooled human plasma was purchased from George King Biomedical Inc (Overland Park, KS, USA). Individual human semen and plasma from normal healthy donors were purchased from BioIVT (Westbury, NY, USA).

### Clinical study and samples used for kinetic evaluation of AAV5 vector capsids

Plasma and semen samples from hemophilia A participants treated with AAV5-hFVIII-SQ in a Phase 1/2 dose-escalation study (270-201, CTID NCT02576795, EudraCT 2014-003880-38) were evaluated using the iqPCR assays. Patients with severe hemophilia A and no pre-existing immunity to AAV5, as measured by transduction inhibition and total antibody assays, were enrolled in the study. Participants were enrolled into one of four dose cohorts including, 6E12 vg/kg (Cohort 1, *n* = 1), 2E13 vg/kg (Cohort 2, *n* = 1), 6E13 vg/kg (Cohort 3, *n* = 7), and 4E13 vg/kg (Cohort 4, *n* = 6). Samples used for iqPCR were collected on day 4, day 8 (week 1), and then either weekly until week 16 or biweekly until week 16 (starting at week 4), and monthly or bimonthly thereafter. Samples were tested in iqPCR until three consecutive negative results (below the limit of detection, BLD) were obtained. Analysis of AAV5 capsid shedding kinetics included evaluation of peak capsid concentration, time-to-peak capsid concentration, time of last positive visit, and time to first negative visit confirmed by two consecutive negative visits. Capsid levels reported as BLQ were imputed as one-half of the LLOQ for kinetic analysis and graphical display, based on classical pharmacokinetics methodology.

### Semen pre-treatments

To reduce semen viscosity [26], undiluted semen samples were thawed at 37 °C for 5−20 min and diluted 1:3 in PureSperm™ Buffer (Nidacon, catalog no. PSB-100, Mölndal, Sweden), followed by vortexing. Liquefaction was performed at 37 °C for 30−60 min without rocking or shaking. Samples were then frozen at −60 °C or colder and thawed at 37 °C for a total of three freeze/thaw cycles to lyse cells within the semen matrix.

### iqPCR-based AAV5 capsid detection

Given that serum samples from hemophilia A participants can be difficult to procure, most clinical samples had been collected as plasma. Thus, standard curves were prepared in plasma using a 1:10 dilution of AAV5-FVIII-SQ in undiluted plasma, followed by additional 1:10 dilutions to generate seven non-zero calibrators (2.08E+10 to 2.08E+04 vg/mL). Similarly, standard curves in semen were prepared using a 1:30 dilution of AAV5-FVIII-SQ in liquefied semen (MRD3), followed by additional 1:10 dilutions to generate seven non-zero calibrators (2.08 E+10 to 2.08E+04 vg/mL). Calibrator 2.08E+04 vg/mL was an anchor point. A negative control matrix without an analyte was included in every experiment. Standard curves consisted of six non-anchor calibrator points: 2.08E+10 (STD01, ULOQ), 2.08E+09 (STD02), 2.08E+08 (STD03), 2.08E+07 (STD04), 2.08E+06 (STD05), and 2.08E+05 (STD06, LLOQ) vg/mL. An additional calibrator point at 2.08E+04 vg/mL (STD07) served as an anchor point. Standards, controls, and samples were added to 96-well round-bottom non-binding plates (VWR catalog no. 43047). In the plasma assay, 90 μL of undiluted spiked and unspiked plasma samples were mixed with 90 μL of 1.5 μg/mL biotinylated anti-AAV5 capture antibodies (clone ADK5a, Progen, catalog no. 615148, Heidelberg, Germany) in Tris-buffered saline with Tween (TBST) for 1−2.5 h at ambient temperature; in the semen assay, 60 μL of liquefied semen (see above) were mixed with 60 μL of 50U/100uL Benzonase (MilliPore Sigma catalog no. 1016950001, Burlington MA, USA) in PBS at 37 °C for 30−60 min, followed by adding 60 μL of 2.25 μg/mL biotinylated anti-AAV5 antibodies for 1−2.5 h at ambient temperature. The addition of Benzonase to semen samples was necessary to hydrolyze cellular nucleic acids and reduce cell lysate viscosity. All subsequent plate incubation steps were performed identically for both matrices, at 300 rpm shaking; samples and reagent preparations were vortexed briefly before use. Once antibody-capsid complexes were formed, 25 μL of 1.6 mg/mL streptavidin-coated magnetic beads (Invitrogen, catalog no. 65001, Carlsbad, CA, USA) were washed three times with phosphate buffer saline (PBS) and added to samples for 1−2 h. Plate magnets were used to pellet beads, and supernatants were removed by three washes with PBS. Benzonase in PBS prepared at 5U/100uL (plasma) or 50U/100uL (semen) was added to the plate for 15−45 min at 37 °C to digest all residual forms of single, double, linear, or circularized DNA that may have remained non-specifically bound to the external surface of the AAV5 capsid [12]. After one wash with PBS, 25 μL (plasma assay) or 20 μL (semen assay) of Elution Buffer (Qiagen, EB Buffer catalog no. 1014609, Hilden, Germany) were used to resuspend the beads, thereby concentrating the analyte from the original sample volume. The plate was heated to 60 °C for at least 10 min to denature the AAV5 capsids and release the encapsidated vector DNA. Beads were pelleted and supernatants were tested by qPCR on a Roche Light Cycler 480 II to detect the encapsidated FVIII-SQ vector DNA. PCR conditions were identical to those described below for qPCR.

### Limit of detection (LOD) determination for iqPCR

Plasma and semen samples with known AAV5-FVIII-SQ concentrations ≤ LLOQ were prepared and processed using five independent immunoprecipitations. At least 10 µL from each of the five immunoprecipitations were pooled per concentration level, aliquoted, and tested as ten identical replicates in qPCR. The LOD was statistically determined by a logistic regression model in JMP using transformed Cp values. The Cp value for each concentration of AAV5-FVIII-SQ was transformed into detectable or undetectable and given a value of 0 or 1, respectively. If a well had value 0 < *x* < 40, it was given a transformed value of 0, or detectable. If the well had a value equal to 0 or 40 it was given a transformed value of 1, or undetectable. The transformed values were then imported into JMP version 12 and the column labeled ‘detectability’ and plotted against nominal vg/mL using the fit model analysis in JMP. Detectability (*y*-value) was labeled as a nominal value, while AAV5-FVIII-SQ concentration in vg/mL (*x*-value) was labeled as a continuous value. Upon running the logistic regression analysis, an equation was calculated to fit the data and used to calculate the probability that a certain capsid concentration was detectable. An inverse prediction plot was then used to back-calculate the capsid concentrations at varying probabilities of detection. The LOD was derived as the AAV5-FVIII-SQ capsid concentration (vg/mL) in neat plasma or semen with a 95% probability of detection, implying that this concentration would be detected in 95 of 100 replicate wells. The 95% confidence limits for the LOD were also reported.

### qPCR detection of FVIII-SQ vector DNA

Twenty microliter PCR reactions were prepared in duplicate that contained 5 μL sample and 15 μL of master mix consisting of 10 μL environmental mix (Life Technologies, catalog no. 4396838, Carlsbad, CA, USA) and 2.8 μL of 900 nM of forward and reverse primers, and 250 nM of fluorescent probes targeting FVIII-SQ (Eurofins Genomics, Louisville, KY, USA), and 2.2 μL of DNA/RNAse-free water. The sequences for primers and probe were as follows: forward primer: 5′-ATGCACAGCATCAATGGCTA-3′; reverse primer 5′-CCATCTTGTGCTTGAAGGTG-3′; and probe FAM-CCTGAGCATTGGGGCCCAGA-BHQ1. Following denaturation at 95 °C for 10 min, 45 thermal cycles were performed at 95 °C for 15 s and at 60 °C for 60 s. Fluorescent signals at 465–510 nm were used to detect the FVIII-SQ amplicon on Roche Light Cycler 480. Quantitative cycle cross-point values (Cp values) were derived from a determination of the amplification cycle at which the product amplification curve makes the sharpest change in slope, which is also known as the second derivative maximum of the amplification curve (Light Cycler 480 software version 4). This Cp value is indirectly proportional to the amount of DNA starting material, allowing for back-calculation of vector genomes in unknown samples based on the standard curve. To construct the standard curve, raw C_p_ values from all seven non-zero calibrators were used to generate a linear regression, and the concentration of each replicate per calibrator was back-calculated using the following linear equation: Cp = slope * log_10_ (concentration [vg/mL]) + *y*-intercept. The mean back-calculated concentration (BCC), CV and RE were reported for each calibrator point. The slope and *R*^2^ values were also reported for each curve. The LOD for qPCR was statistically determined by logistic regression using transformed Cp values, similar as described for iqPCR but using FVIII-SQ DNA concentration in vg/reaction as the continuous variable. The LOD was derived as the quantity of FVIII-SQ DNA (vg/reaction) with a 95% probability of detection. The LOD was then back-calculated from vg/reaction to vg/mL in plasma or semen, using matrix-specific conversion factors that accounted for dilution steps and volume changes during the assay.

### Cell-based AAV5 transduction assay

HepG2 cells (VWR catalog no. MSPP-HB8065, Radnor, PA, USA) were plated at 400,000 cells/well in a 24-well plate for 24 h, allowing enough time for the cells to attach to a culture plate as a monolayer. AAV5-FVIII-SQ was spiked into human plasma at various concentrations, which served as surrogate test samples during assay development. Twenty-five microliters of each test sample was added to one well with HepG2 cells containing 100 µl CPC-1 (Lonza, catalog no.77232, Basel, Switzerland) culture media. The plate was then placed in a moisture-maintaining container (to avoid media evaporation) and incubated at 37 °C overnight. On the following day, 125 µL of a 200 µM etoposide solution (VWR catalog no. 89158-868) was added to each well (final volume of 250 µL) to enhance transduction efficiencies. For assay readout, cell culture supernatant was harvested at Day 14 after transduction and analyzed for the presence of FVIII activity using a Chromogenix Coatest SP4 Factor FVIII kit (Diapharma, catalog no. K824094, West Chester, OH, USA), following the manufacturer’s instructions with minor modifications required for a 96-well plate format. Briefly, 25 µL neat culture supernatant or control samples were mixed with 50 µL phospholipid + FIXa + FX and incubated at 37 °C for 4 min. Twenty-five microliters of CaCl_2_ were added and the sample was incubated at 37 °C for 10 min, followed by adding 50 µL substrate solution with a thrombin inhibitor (S-2765 + I-2581) and incubating at 37 °C for 10 min. At last, 25 μl of 20% acetic acid was added at room temperature and the plate was read at 405 nm (OD405) and 490 nm (OD490). Corrected OD405 data were reported after subtracting the OD490 absorbance values. To determine the LOD, supernatant from non-transduced HepG2 cells was used as a negative quality control (NQC), and the OD405 measurements from the transduced cells were expressed as the signal-to-noise (S/N) ratio over the OD405 measurement from the non-transduced cells. Thus, the S/N ratio served as a relative measure for the FVIII activity levels in cell supernatants after AAV5-FVIII-SQ transduction. The cutoff for positive detection was set at S/N ≥ 1.5. The LOD was then reported as the lowest concentration of AAV5-FVIII-SQ (vg/mL) in neat plasma observed for a positive test sample.

### ELISA-based AAV5 capsid detection (immunoassay 1)

A commercial ELISA kit (Progen catalog no. PRAAV5) was modified for use with an AAV5-FVIII-SQ standard curve in human plasma. AAV5-FVIII-SQ in formulation buffer was diluted 1:10 in neat plasma, followed by serial 1:4 dilutions in kit buffer with 10% pooled plasma matrix (corresponding to MRD 10 for test samples) prior to plating. The kit buffer containing 10% plasma without analyte was plated as the negative control. The manufacturer’s instructions were followed for reagent preparation and procedures, using proprietary 96-well plate strips pre-coated with anti-AAV5 capture antibodies (ADK5a). AAV5-FVIII-SQ captured from standards and test samples was detected using a biotinylated anti-AAV5 detection antibodies (ADK5a) and a streptavidin-peroxidase conjugate. Substrate addition generated a colorimetric signal measured at 405 nm that was directly proportional to the amount of AAV5 capsids in the sample.

### Electro-chemiluminescence-based (ECLA) AAV5 capsid detection (immunoassay 2)

Meso Scale Discovery (MSD, Kenilworth, NJ, USA) was the platform used for the electro-chemiluminescence assay (ECLA). Undiluted human plasma was used to prepare an AAV5-FVIII-SQ standard curve and QCs. Undiluted plasma samples, standards, and QCs were added to a 96-well round-bottom non-binding plate (VWR catalog no. 43047). Biotinylated anti-AAV5 capture antibodies (ADK5a, Progen catalog no. 615148) at 1.0 and 20 ng/mL ruthenylated anti-AAV5 detection antibodies (ADK5b, Progen catalog no. 610149) were first mixed in TBST, and then added 1:1 to samples on the plate. The ruthenylated detection antibody was labeled in-house at 2 mg/mL in PBS with 10 nmol Sulfo-tag at a challenge ratio 1:12. During antibody-antigen complex formation, a streptavidin-coated plate (MSD catalog no. L15SA-1) was blocked with 6% Bovine Serum Albumin (BSA) in TBST. After 1−2 h at ambient temperature with 400 rpm shaking, antibody-antigen complexes were added to blocked and PBST washed MSD plates for 1 h, and after washing, 1× MSD Read Buffer T (MSD catalog no. R92TA-2) was added. The plate was read on MSD QuickPlex 2400. Measured electro-chemiluminescent signals are directly proportional to the amount of AAV5 capsids in the sample.

### Single-molecule counting (SMC)-based AAV5 capsid detection (immunoassay 3)

SMC was performed on the Singulex^®^ Erenna^®^ instrument (MilliPore Sigma). Biotinylated anti-AAV5 capture antibodies (Progen, Cat no. 615148) and proprietary Erenna^®^ labeled anti-AAV5 detection antibodies (ADK5b) were diluted in TBST and added to test samples, standards, and controls (diluted to MRD 4), Samples were subsequently captured using streptavidin-coated beads (Invitrogen catalog no. 65001) and a magnetic plate. After washing, proprietary elution buffer was added at 20% of the original sample volume. On the Erenna^®^ instrument, samples were taken up by a narrow capillary where antibodies passed through an interrogation space for single-molecule counting using a fluorescent signal. Event Photons (EPs; low end and mid-level signal) were used for reporting and the signals measured were directly proportional to the amount of AAV5 capsids in the sample.

### LOD determination for immunoassays

LODs were determined using normalized signal-to-noise ratios (S/N) from standard curves. Raw assay signal values from all non-zero calibrators were divided by the signal of the negative control to calculate S/N ratios. The cutoff for positive detection was set at S/N ≥ 1.5. The LOD was then reported as the lowest concentration of AAV5 capsids (vg/mL) in neat plasma observed for a positive test sample.

### AAV5 TAb assay for human plasma and human semen

TAb against AAV5 was measured in human plasma or semen using a sequential bridging ECLA on the Meso Scale Discovery (MSD) platform, using a previously published method [27]. All plate incubation steps were performed for 1 h with shaking at ambient temperature, followed by washing with TBST. Standard-bind Multi-Array MSD plates were coated with 6.65 µg/mL of AAV5 in PBS and blocked with TBS-C. QCs were prepared in 100% pooled human cutpoint-control (CC) plasma or semen using a rabbit polyclonal anti-AAV5 antibody at 75 ng/mL (LQC) and 14.1 µg/ml (HQC). In the plasma assay, test samples, QCs, and pooled plasma CC were diluted to the minimally required dilution (MRD) of 1:20 in TBS-C (screening assay) and added in duplicate to the plate. In the titer assay, samples were serially diluted 1:3 in neat pooled plasma CC, followed by dilution to MRD 20, and tested as in the screening assay. In the semen assay, test samples, QCs, and pooled semen CC were first diluted 1:5 in PureSperm Buffer and incubated at 37 °C for 30−60 min, then diluted 1:4 in TBS-C with 50U/0.1 mL of Benzonase and incubated for 15 min at 60 °C (final MRD 20). In the titer assay, samples were serially diluted 1:3 in liquefied pooled semen CC prior to the 1:4 dilution and tested as in the screening assay. Serially diluted HQC served as a titer quality control (TQC) in both the plasma and semen assay. For detection, 1.0 μg/mL ruthenium-labeled AAV5 capsid in TBS-C was added, and after the addition of 2 × MSD Read Buffer T, electrochemiluminescence was detected by the MSD MESO QuickPlex SQ 120 (Imager) and Meso Scale Discovery Workbench version 4.0.12. Sample results were generated using Watson LIMS version 7.6 and expressed as positive if above the assay cutpoint, negative if below, and with numerical titer values reported as applicable.

## Results

### Limit of detection (LOD) and comparison of assay sensitivities

**Fig. 1:**
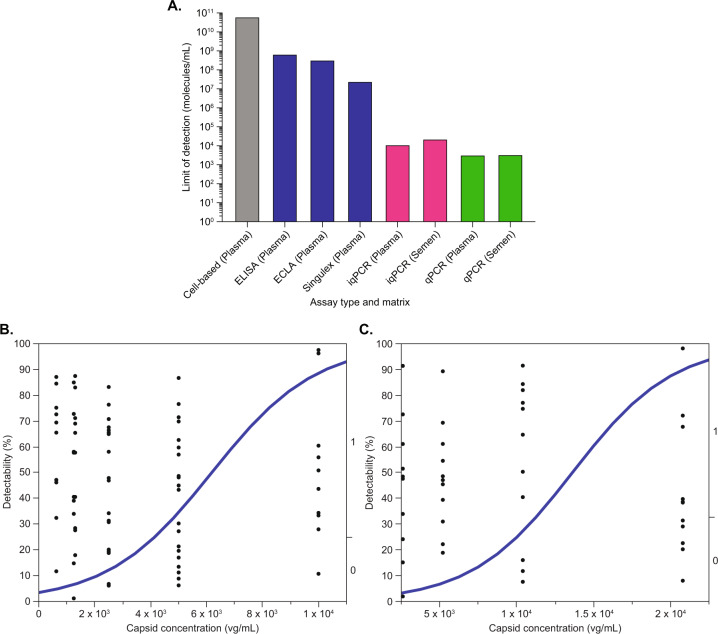
Evaluation of assay sensitivity. Comparison of LODs between different capsid detection methods (**A**): cell-based transduction assay (grey bars), immunoassays (blue bars), iqPCR (pink bars), and conventional qPCR (green bars). Detectability (fixed binary variable) of individual sample replicates (black dots) in neat plasma (**B**) and semen (**C**) was plotted against the nominally spiked AAV5-FVIII-SQ concentration in vg/mL (continuous variable). Dots below the logistic regression curve (blue line) were detectable, dots above the curve were undetectable. Logistic regression analysis was performed to calculate an equation to fit the data, based on the probability of detection. The inverse prediction was used to determine LOD, defined as the AAV5-FVIII-SQ concentration with a 95% probability of detection.

To monitor the clinical presence of AAV5-FVIII-SQ vector capsids, we first assessed the sensitivity of several existing AAV5 capsid detection methods in human plasma, including a cell-based transduction assay and three different capsid immunoassays (ELISA, ECLA, and SMC). None of these methods achieved sufficient sensitivity when compared to conventional qPCR (Fig. 1A). The transduction assay, ELISA, ECLA, and SMC assays had LODs of 6.40E+10, 7.01E+08, 3.42E+08, 2.50E+07 capsids/mL in plasma, respectively. In sharp contrast, conventional qPCRs that measure total FVIII-SQ vector DNA in extracted plasma and semen specimens had LODs of 3.32E+03 and 3.49E+03 vg/mL, respectively.

Given these data and the expected low capsid levels in clinical samples, there was a need to develop a more sensitive approach that would approximate the sensitivity offered by conventional qPCR. To this end, we established an iqPCR method by combining AAV5 capsid immunocapture from plasma or semen, followed by qPCR detection of encapsidated vector DNA. The efficiency of the immunocapture step was similar in plasma and semen and ranged from 1.4 to 6.3%. The LOD for iqPCR in each matrix was defined as the minimum concentration of AAV5-FVIII-SQ vector capsid that was reliably detectable, but not necessarily quantifiable. To assess the LOD, plasma and semen samples with decreasing concentrations of AAV5-FVIII-SQ were prepared and tested in 10 qPCR replicates. LOD for plasma was tested in two independent experiments, and LOD for semen was tested in one experiment. For plasma, data from both experiments were combined and analyzed by logistic regression to determine the minimum quantity of AAV5-FVIII-SQ that can be reliably detected with 95% consistency (LOD_95_). The LOD_95_ was calculated as 1.17E+04 vg/mL of AAV5-FVIII-SQ in neat plasma, with 95% confidence interval (CI) limits of 9.05E+03 and 1.87E+04 vg/mL (Fig. 1B). For semen, the LOD_95_ was calculated as 2.33E+04 vg/mL of AAV5-FVIII-SQ in neat semen, with 95% CI limits of 1.83E+04 and 3.88E+04 vg/mL (Fig. 1C). Hence, the LOD for iqPCR was at least three orders of magnitude lower than those for other capsid detection methods, approaching the sensitivity of conventional qPCR methods (Fig. 1A). These results demonstrate analytically suitable sensitivity of iqPCR for detecting ultra-low AAV5 capsid levels in human biofluids.

### Standard curve performance

Standard curve performance was monitored over the entire iqPCR method characterization to demonstrate adequate curve fit, slope, and acceptable precision and accuracy of each calibrator point. For plasma, in total, 13 of 17 experiments had standard curves that met acceptance criteria, with *R*^2^ values > 0.995, and slopes of the linear regression curve between −3.54 and −3.31 (Table 1, top). The inter-curve relative error (RE) was −8.6 to +5.0% and the inter-curve coefficient of variation (CV) ≤ 14.3% for all non-anchor calibrator points. The intra-curve RE was −38.6 to +42.9% and the intra-curve CV ≤ 23.4% for all non-anchor calibrator points. Overall, these data demonstrate acceptable standard curve performance in about 80% of all experiments. For semen, five of seven total experiments had standard curves that met target acceptance criteria, with *R*^2^ values > 0.984, and the slopes of the linear regression curve between −3.57 and −3.37 (Table 1, bottom). The inter-curve RE was −14.7 to +29.3%, and inter-curve CV ≤ 26.3% for all non-anchor calibrator points. The intra-curve RE was −45.9 to +92.1%, and intra-curve CV ≤ 28.4% for all non-anchor calibrator points. Overall, these data demonstrate acceptable standard curve performance in about 70% of all experiments.

### Precision, accuracy, and range of quantification

Accuracy and precision were evaluated to demonstrate the level of consistency for quantitative test sample results within and between assays. Five quality controls (QCs), including a lower limit of quantification control (LLOQ, 2.08E+05), low-quality control (LQC, 2.08E+06), medium quality control (MQC, 2.08E+07), high-quality control (HQC, 2.08E+09), and upper limit of quantification control (ULOQ, 2.08E+10) were prepared by spiking AAV5-FVIII-SQ vector into pooled plasma or semen. Plasma was tested in three replicates per iqPCR experiment over four experiments, conducted by two analysts on four different days. One of three LLOQ replicates in Experiment 1 was approximately 1-log higher than the nominal concentration (1.66E+06 vg/mL vs. 2.08E+05 vg/mL) and was removed as a statistical outlier by Q-test (Table 2, left). For the remaining plasma samples, the RE ranged from −6.6 to +8.0%, intra-assay CV was ≤16.1% and intra-assay total errors (TE) were ≤20.9%. The inter-assay RE ranged from −6.6 to +10.4%, inter-assay CV was ≤28.9% and inter-assay TE were ≤39.3%.

Semen was tested in three replicates per by iqPCR experiment over three experiments, conducted by two analysts on three different days. For all QCs excluding the LLOQ, the intra-assay RE ranged from −11.0 to +6.6%, intra-assay CV was ≤9.6% and intra-assay TE was ≤18.9% (Table 2, right). The inter-assay RE ranged from −11.0 to +6.6%, inter-assay CV ≤ 14.9%, and inter-assay TE ≤ 25.8%. For the ULOQ, in one of three experiments, all three replicates were above the target acceptance range for accuracy (REs between +107.8 and +123.2%). This also led to variable inter-assay precision (CV ≤ 60.4%); however, inter-assay RE (target criteria of −50 to +100%) and inter-assay TE (target criteria of ≤130%) for the ULOQ were +60.0 and 120.3%, respectively, and thus remained within acceptance range.

In summary, these data demonstrate acceptable precision and accuracy across the range of quantification from 2.08E+05 to 2.08E+10 vg/mL AAV5 vector capsid in neat plasma, and from 2.08E+05 to 2.08E+09 vg/mL AAV5 vector capsid in neat semen.

### Assessment of specificity and interference

**Fig. 2:**
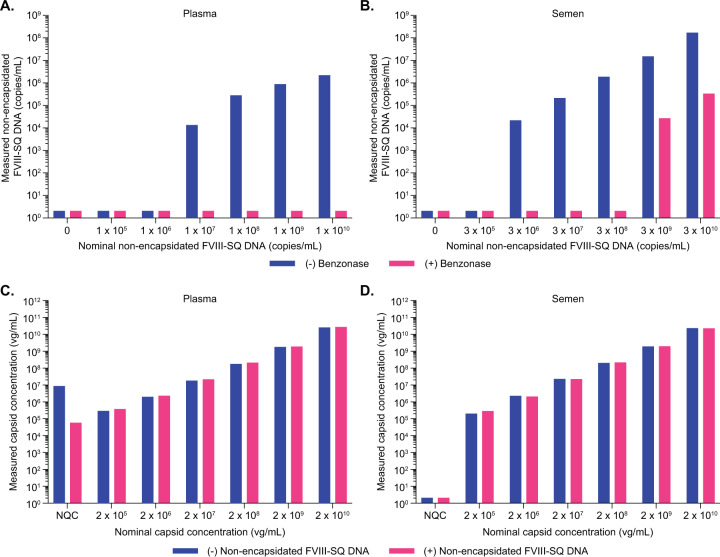
Assessment of assay specificity. Plasma (**A**) and semen (**B**) samples were spiked with increasing amounts of non-encapsidated FVIII-SQ DNA and tested in iqPCR either under standard assay conditions that included a Benzonase digest (pink bars) or without Benzonase (blue bars). Plasma (**C**) and semen (**D**) samples were spiked with increasing amounts of AAV5-FVIII-SQ capsids and co-spiked with a fixed amount of non-encapsidated FVIII-SQ DNA (plasma: 5E+09 vg/mL; semen: 1E+09 vg/mL) and tested in iqPCR.

Plasma and semen samples collected after AAV5 gene therapy administration may contain non-encapsidated vector DNA. To exclude potential cross-reactivity for iqPCR, a serial dilution of non-encapsidated vector DNA (using a FVIII-SQ plasmid) was prepared ranging from 1E+10 to 1E+05 vg/mL in plasma and tested in iqPCR. The same material was diluted from 3E+10 to 3E+05 vg/mL in semen and tested in iqPCR. No response above the LOD was observed for up to 1E+10 vg/mL non-encapsidated vector DNA in plasma (Fig. 2A, pink bars), and for up to 3E+08 vg/mL in semen (Fig. 2B, pink bars). Non-encapsidated vector DNA tested at 3E+09 vg/mL in semen was above the LOD but remained below the limit of quantification (BLQ). Specificity was critically dependent on the inclusion of the Benzonase step after the immuno-capture of the capsid, since non-encapsidated vector DNA did generate a low positive response at and above 1E+07 vg/mL in plasma and 3E+06 vg/mL semen, if the Benzonase step was omitted (Fig. 2A, B, blue bars).

To further demonstrate that non-encapsidated vector DNA did not interfere with the quantification of AAV5 vector capsids by iqPCR, samples were spiked with 5E+09 vg/mL (plasma) or 1E+09 vg/mL (semen) non-encapsidated vector DNA (FVIII-SQ plasmid) and co-spiked with AAV5-FVIII-SQ vector capsids at NQC and increasing concentrations in matrix. As a control, the same samples were also tested without non-encapsidated vector DNA. All plasma samples (Fig. 2C) and semen samples (Fig. 2D) containing AAV5-FVIII-SQ vector capsids remained within target acceptance criteria for accuracy (RE ranged from −9.0 to +47.3%) compared to nominal spike levels, regardless of the presence or absence of non-encapsidated vector DNA. Thus, the presence of up to 5E+09 vg/mL non-encapsidated vector DNA in plasma and up to 1E+09 vg/mL non-encapsidated vector DNA in semen did not interfere with the quantification of AAV5 vector capsids. For plasma, the NQC was above the LOD (though remained below the limit of quantification, BLQ) in the presence of 5E+09 vg/mL non-encapsidated FVIII-SQ vector DNA (Fig. 2C). However, the NQC also showed detectable capsid levels in the absence of non-encapsidated FVIII-SQ DNA, rendering all NQC results difficult to interpret in this experiment. Therefore, the data obtained for non-encapsidated vector DNA without co-spiked AAV5-FVIII-SQ vector capsids described above (Fig. 2A) were used to conservatively specify the maximal level of non-encapsidated vector DNA tolerated by iqPCR as 1E+09 vg/mL. For semen, the NQC was below the limit of detection (BLD) in the presence of 1E+09 vg/mL non-encapsidated vector DNA (Fig. 2D), which was therefore reported as the maximal level tolerated by iqPCR in this matrix.

### Assessment of selectivity

**Fig. 3:**
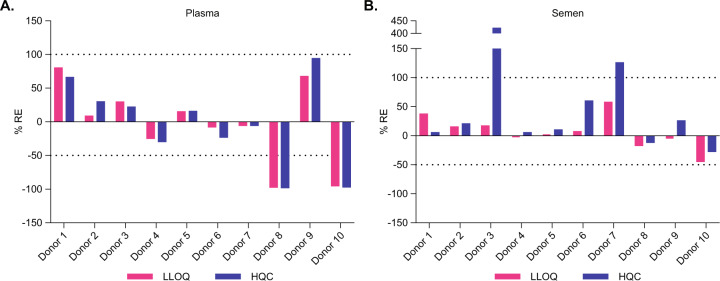
Assessment of selectivity. Plasma (**A**) and semen (**B**) samples from ten individual donors were spiked with AAV5-FVIII-SQ at 2E+05 vg/mL (LLOQ, pink bars) and 2E+09 (HQC, blue bars) and tested in iqPCR. Accurate recovery was assessed by determining the relative error (RE) between measured and nominally spiked capsid concentration. Dotted lines represent the target performance range for RE (−50 to +100%).

Selectivity in plasma was assessed to evaluate whether variations of plasma matrix components in individual donors can impact AAV5 vector capsid detection or quantification. To this end, plasma samples from ten individual donors were left unspiked (NQC) or spiked with AAV5-FVIII-SQ at LLOQ or HQC concentration and tested in iqPCR. 9/10 (90%) unspiked donor samples were BLD, and the remaining unspiked donor sample was positive but remained BLQ (data not shown). 8/10 (80%) LLOQ or HQC-spiked donor samples were within acceptance criteria for quantitative reporting (Fig. 3A), demonstrating overall passing selectivity assessment for plasma matrix (≥80% donors need to pass at each QC level). The remaining two donor samples (donor 8 and 10) were both BLD at LLOQ and under-recovered at HQC levels. An orthogonally performed total AAV5 antibody ECL assay showed that these two donors had pre-existing anti-AAV5 antibodies (data not shown), which likely interfered with the immuno-capture of the AAV5 vector capsid (more information provided in the following section). If these two donors were excluded from the selectivity assessment, 7/8 (87.5%) unspiked donor samples and 8/8 (100%) LLOQ or HQC-spiked donor samples were within acceptance criteria for quantitative reporting. Hence, variations of plasma matrix components other than increased anti-AAV5 antibody levels did not interfere with capsid quantification in the iqPCR assay.

We used the same design to evaluate selectivity in individual semen samples. 8/10 (80%) unspiked donor samples were BLD, and the remaining two unspiked donor samples were positive but BLQ (data not shown). 10/10 (100%) LLOQ-spiked and 8/10 (80%) HQC-spiked donor samples were within acceptance criteria for quantitative reporting (Fig. 3B), demonstrating overall passing selectivity assessment for semen matrix (≥80% donors need to pass at each QC level). The remaining two donor samples (donor 3 and 7) showed over-recovery of vector capsids at HQC level.

### Evaluation of anti-AAV5 antibody interference

**Fig. 4:**
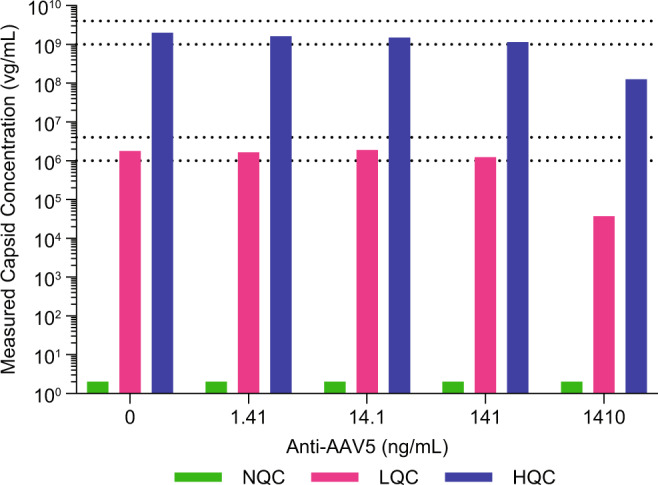
Evaluation of anti-AAV5 antibody interference. Plasma samples were spiked with increasing amounts of anti-AAV5 antibodies and either left unspiked (NQC, green bars) or spiked with AAV5-FVIII-SQ at 2E+06 vg/mL (LQC, pink bars) and 2E+09 vg/mL (HQC, blue bars) and tested in iqPCR. Dotted lines represent the target performance range, based on the capsid concentrations for LQC (bottom range) and HQC (top range) measured in the absence of anti-AAV5 antibodies, and using an RE from −50 to 100%.

As shown during selectivity assessment, pre-existing anti-AAV5 antibodies may interfere with quantification in iqPCR. Since plasma samples collected after AAV gene therapy administration contain high titers of anti-AAV antibodies [28–31], the ability of the iqPCR method to detect and quantify AAV5 vector capsids in the presence of anti-AAV5 antibodies was evaluated in greater detail to better understand potential limitations. To this end, samples spiked with AAV5-FVIII-SQ at NQC, LQC or HQC were co-spiked with increasing concentrations of rabbit polyclonal anti-AAV5 antibodies (1.41, 14.1, 141, and 1410 ng/mL). As a control, the same QC samples were also tested in the absence of anti-AAV5 antibodies. All NQC samples remained BLD; LQC and HQC samples with ≤141 ng/mL rabbit polyclonal anti-AAV5 antibodies were within acceptance criteria (Fig. 4). In contrast, rabbit polyclonal anti-AAV5 antibodies spiked at 1410 ng/mL interfered with the accurate quantification of AAV5-FVIII-SQ, leading to BLQ results for LQC-spiked samples and under-recovery of HQC-spiked samples. Therefore, up to 141 ng/mL polyclonal anti-AAV5 antibodies in plasma can be tolerated by iqPCR. Evaluation of anti-AAV5 antibody interference was not assessed for semen matrix, since antibodies present in this matrix are highly likely to exert a similar degree of interference.

### Clinical kinetics of AAV5-FVIII-SQ vector capsids

**Fig. 5:**
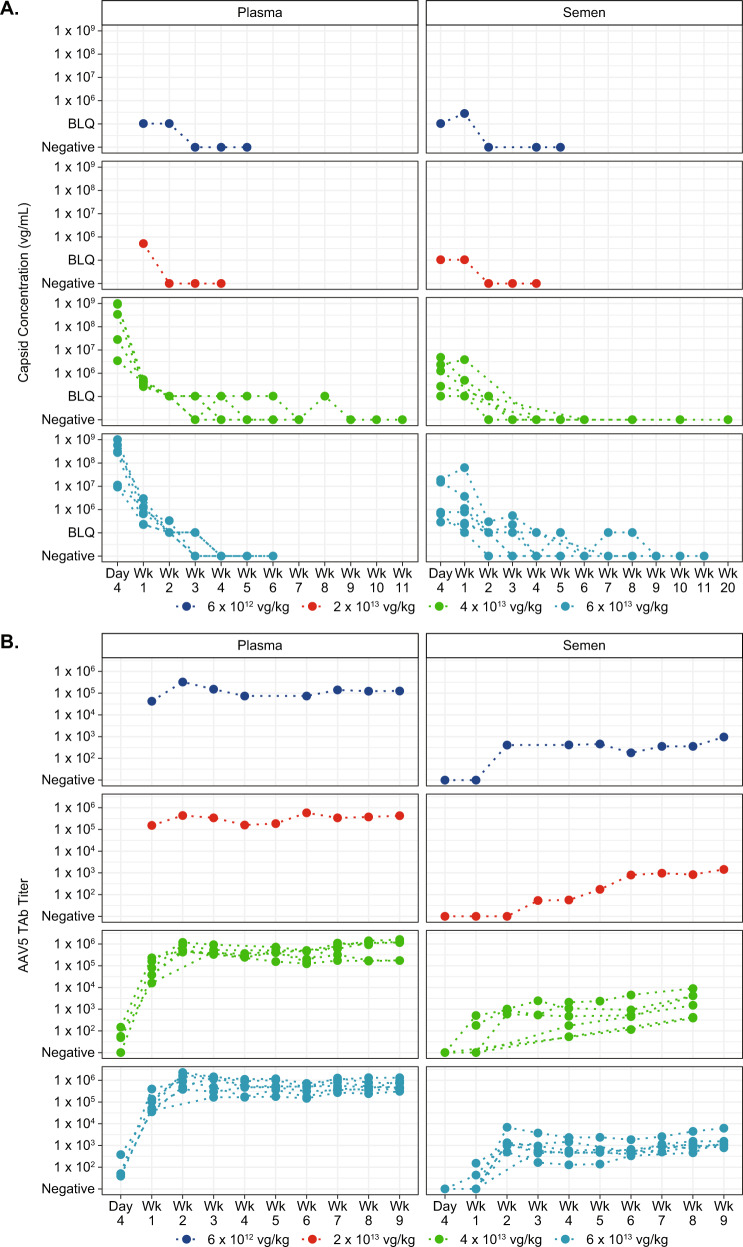
Capsid levels and anti-AAV5 antibody titers in plasma and semen following AAV5-FVIII-SQ administration. Capsid concentrations **A** in plasma (left) and semen (right) from individual participants in clinical study 270-201 were measured by iqPCR following AAV5-FVIII-SQ dose administration at 6E+12 vg/kg (dark blue, *n* = 1 participant), 2E+13 vg/kg (red, *n* = 1 participant), 4E+13 vg/kg (green, *n* = 6 participants), and 6E+13 vg/kg (light blue, *n* = 7 participants) and plotted by visit. Testing of plasma and semen samples was performed for each participant until three consecutive negative results were obtained. Total antibodies to AAV5 **B** in plasma (left) and semen (right) from individual participants in clinical study 270-201 were measured by AAV5 TAb assays using the same dose cohorts as above. Testing of plasma and semen samples was performed for each participant at day 4 if available, and then weekly through week 9.

Based on the method performance characterization described herein (summarized in Table 3), the iqPCR method was considered fit-for-purpose and used to evaluate AAV5 vector capsid levels in a clinical study (270-201) with AAV5-FVIII-SQ (valoctocogene roxaparvovec, BMN 270). Following 6E+12 to 6E+13 vg/kg dose administration, vector capsids were detectable in plasma and semen for all 15 participants (Fig. 5A). Peak capsid levels in plasma were observed shortly after dose administration, with a median time to peak levels across all participants of 0.4 and 0.6 weeks in plasma and semen, respectively (Tables 4 and 5). Median peak capsid levels generally increased with dose. In the single participant who received 6E+12 vg/kg AAV5-FVIII-SQ, the peak capsid concentration was BLQ in plasma and was 2.9E+05 vg/mL in semen. In the 6E+13 vg/kg cohort, the median peak capsid concentration was 2.8E+08 and 7.7E+05 vg/mL in plasma and semen, respectively. Across all participants, the median time to the last positive sample was 2.1 and 1.3 weeks in plasma and semen, respectively. All participants achieved the first of three consecutive negatives samples with a median (min, max) time of 3.1 (2.5, 9.0) and 3.3 (1.8, 9.0) weeks in plasma and semen, respectively.

In parallel, anti-AAV5 total antibody titers were measured in order to evaluate their potential impact on AAV5 capsid quantification. Antibody titers in plasma rose quickly following dose administration and ranged from 1.60E+04 to 3.99E+05 at week 1 (Fig. 5B). Antibody titers were lower in semen, with titers ranging from <MRD to 5.10E+02 at week 1 and from <MRD to 6.86E+03 at week 2. Thus, while it appears likely that some AAV5 capsids complexed by these antibodies may not be fully accounted for in the numerical iqPCR results, these data demonstrate that highly sensitive detection of intact AAV5 capsids was sustained in clinical samples, even in the presence of anti-AAV5 antibodies.

## Discussion

The iqPCR method described herein detects ultra-low levels of residual AAV5 vector capsids in human plasma and semen by using capsid-directed immunocapture followed by qPCR amplification of encapsidated vector DNA. This method was developed to support clinical pharmacokinetics and shedding studies for AAV5-FVIII-SQ (valoctocogene roxaparvovec, BMN 270), but the general assay format can be easily modified for use with other AAV or non-AAV based gene therapies by substituting the antibodies, PCR primers, and probe accordingly.

During iqPCR method characterization (summarized in Table 3), standard curve performance, as well as precision and accuracy of QCs, were acceptable within the established range of quantitation. The resulting lower limit of quantification (LLOQ) for AAV5 vector capsids (2.08E+05 vg/mL), and the statistically derived, reliable limit of detection (LOD_95_, 1.17E+04 vg/mL in plasma and 2.33E+04 vg/mL in semen) offered a substantial improvement over cell-based transduction assays and capsid immunoassays, approaching the sensitivity of conventional qPCR vector shedding assays. Therefore, iqPCR is a sufficiently sensitive, precise, and accurate method to further characterize the nature of residual vector DNA detected in human biofluids by conventional qPCR. The advantage of using iqPCR is the enhanced ability to evaluate the potential transmission risk, given the method’s specificity for DNA contained in structurally intact AAV vector capsids.

We used a broader than usual range of acceptable accuracy for iqPCR (−50 to +100% RE), based on the following arguments: I) PCR amplification is exponential, and Cp raw values are acquired using a logarithmic (log_2_) scale. Consequently, a decrease in Cp value by 1 corresponds to doubling of capsid quantities (relative change: +100%), while an increase in Cp value by 1 corresponds to halving the quantities (relative change: −50%). Therefore, a target RE range of −50 to +100% for capsid quantities will ensure that Cp raw data accuracy falls within ±1 qPCR cycle. This is similar to target acceptance criteria used to specify the accuracy required for antibody titers, which is ±1 log_2_(titer) if obtained using a 1:2 dilution scheme, corresponding to the last detectable sample dilution shifting one step up or down. Using these RE criteria for iqPCR, raw Cp values larger than 10 (typical measurement range) are required to remain within at least 10% accuracy. II) Since AAV capsids containing vector DNA are first immuno-precipitated from plasma and then quantified by qPCR, there is an additional source of analytical variability that needs to be accounted for when setting overall acceptance criteria for this two-step assay format. III) Lastly, there is only limited impact on the clinical significance of iqPCR results using this broader accuracy range, since the key kinetic parameter for clinical capsid measurements is the duration of detectability rather than absolute quantification. Furthermore, the decline in clinical capsid concentrations is typically analyzed on a log_10_ scale, for which the use of a two-fold RE range appeared reasonably accurate. Taken together, these arguments justified using a broader −50 to +100% RE range for iqPCR.

Similar to the iqPCR method reported herein, previous immuno-PCR methods also demonstrated better sensitivity than corresponding traditional immunoassays [23, 24, 32–34]. The use of beads, rather than plates for antibody-mediated capsid capture in iqPCR also created an opportunity to perform an elution step. This allowed for additional capsid purification prior to elution, using a Benzonase digest of any unprotected vector DNA, rather than detecting the totality of vector DNA present in the immunocapture mix of individual plate wells. Benzonase is an endonuclease that degrades all forms of linear, circular, single, and double-stranded DNA that may be present freely in solution, non-specifically bound to the outer surface of the capsid, or alternatively, reside within structurally damaged capsids [35]. These features, together with the enrichment of AAV5 capsids during the immunocapture step, likely explain the high specificity of iqPCR for encapsidated and thus potentially transduction-competent vector DNA and the high tolerance for interfering nucleic acids.

Recent advances in the sensitivity of AAV capsid ELISA methods were achieved by using a high-affinity AAV receptor (AAVR) for the capture and detection of AAV2 capsids [36]. This method (termed VIRELISA) has the potential to serve as a generic detection platform for multiple AAV serotypes because AAV1 capsids were also detectable. The LOD for AAV2 capsids in VIRELISA was 5E+04 vg/100 microliter test volume, corresponding to 5E+05 vg/mL, in buffered aqueous solution. The LOD in human plasma has not yet been established but is anticipated to be higher than in aqueous solution due to matrix effects. Thus, the iqPCR method described herein remains at least 40 times more sensitive for detecting AAV capsids in human plasma than VIRELISA that presumably is the most advanced ELISA-based capsid method available to date. In addition, iqPCR only detects AAV capsids if they contain specific vector DNA, while VIRELISA indiscriminately detects full and empty AAV vector capsids as well as wildtype AAV capsids of various serotypes that may be present in clinical samples due to natural infections. Hence, iqPCR is not only more sensitive but also more specific than VIRELISA when measuring potentially transduction-competent vector loads in patients following AAV gene therapy.

One caveat of the iqPCR method is its relatively low tolerance for anti-AAV5 capsid antibodies. Thus, iqPCR may underestimate the total level of AAV5 capsids present in clinical test samples, if they contain more than 141 ng/mL anti-AAV5 antibodies, a quantity that corresponds to an estimated titer of 93 in BioMarin’s AAV5 Total Antibody (TAb) assay [27]. In our clinical study, anti-AAV5 antibodies in plasma reached high titers expected to interfere in iqPCR in all tested participants during the first week after dosing. Antibody titers were lower in semen, but expected iqPCR interference levels were also reached within two weeks in all but one tested participant. Nonetheless, AAV5 capsids remained detectable in both matrices up to 8 weeks. This leads us to conclude that even high anti-AAV5 antibody titers do not necessarily completely block the detection of AAV5 capsids using iqPCR, likely due to successful competition of the monoclonal anti-AAV5 antibody (clone ADK5a) used for immunocapture. It remains probable though that the fraction of AAV5 capsids complexed by anti-AAV5 antibodies may be under-estimated. An alternative mechanism  could be that these antibodies directly enhance clinical capsid clearance, for example through uptake by immune cells.

Interference from anti-AAV5 antibodies was not unexpected for iqPCR, considering the use of a monoclonal anti-AAV5 antibody for capsid immunocapture. Similar interference would also be expected for capsid immunoassays, cell-based transduction assays, and VIRELISA. Nonetheless, AAV-specific post-dose antibodies are also known to be highly neutralizing [28–30]. Therefore, accounting for AAV capsids that are complexed with high-titer neutralizing antibodies may in fact overestimate the potential transmission risk. Instead, it may be more clinically meaningful to rely on the quantification of “free” AAV vector capsids, i.e., those that are not bound by AAV antibodies and thus could potentially contribute to horizontal transmission. This “free” portion of AAV vector capsids can be reliably detected by the iqPCR method with high sensitivity, specificity, selectivity, accuracy, and precision. For this reason, no efforts have been made to mitigate antibody interference in iqPCR, even though this could be a valuable objective for future research.

An alternative for distinguishing vector DNA contained in AAV capsids from that present in transduced cells would be to simply remove all cells from a test sample before performing the qPCR steps. While this approach may remove some non-encapsidated DNA, it does not directly demonstrate that the remaining vector DNA in the cell-free matrix is in fact encapsidated; it could also be in the form of naked DNA debris. There is also the concern that some cells, such as platelets or sperm cells, are rather difficult to remove with 100% efficiency from their respective plasma. Finally, there could also be intact AAV capsids inside of cells, which may be useful to include in comprehensive shedding assessments.

When iqPCR was utilized in a clinical study that administered up to 6E+13 vg/kg of AAV5-FVIII-SQ gene therapy to hemophilia A participants, intact vector capsids were observed in plasma and semen but steadily decreased to undetectable levels in all tested participants by week 9. Capsid peak levels in both matrices occurred within the first week after dose administration; the residual capsid levels after the first week were at least 1000 times lower than the quantity of vector capsids minimally required to establish measurable FVIII activity in a cell-based transduction assay (Fig. 1). Therefore, these capsid levels are not expected to exert any biologically meaningful effects.

In addition, the capsid levels measured by iqPCR in plasma and semen were generally in line with the low levels of total vector DNA in these matrices, as measured by conventional PCR in this study and across other AAV gene therapy trials in hemophilia [1–3]. Hence, most of the total vector DNA detected by conventional PCR shortly after dosing is likely to be encapsidated. The value of performing iqPCR, however, stems from the shorter clearance profile for AAV5 capsids compared to that of total vector DNA, the latter of which can be detected by conventional PCR sometimes for many months after dose administration, likely due to its presence in transduced cells. Thus, iqPCR provides advantages for assessing the duration of any risk mitigation strategies deemed necessary to limit potential horizontal transmission. Considering the low capsid levels, together with the replication-incompetent nature and infrequent integration of recombinant AAV vectors [37, 38], the potential impact of horizontal transmission to untreated individuals or release into the environment is generally considered to be minimal. In summary, our data demonstrate the clinical relevance of applying a formally characterized iqPCR method to evaluate residual capsid levels in human biofluids following AAV gene therapy.

## Data Availability

The de-identified individual participant data that underlie the results reported in this article (including text, tables, figures, and appendices) will be made available together with the research protocol and data dictionaries, for non-commercial, academic purposes. Additional supporting documents may be available upon request. Investigators will be able to request access to these data and supporting documents via website (www.BioMarin.com) beginning 6 months and ending 2 years after publication. Data associated with any ongoing development program will be made available within six (6) months after approval of relevant product. Requests must include a research proposal clarifying how the data will be used, including the proposed analysis methodology. Research proposals will be evaluated relative to publicly available criteria available at www.BioMarin.com to determine if access will be given, contingent upon execution of a data access agreement with BioMarin Pharmaceutical Inc. Materials and protocols will be distributed to qualified scientific researchers for non-commercial, academic purposes. The AAV5-hFVIII-SQ vector and the AAV5-hFVIII-SQ vector 468 sequence are part of an ongoing development program, and they will not be shared.
